# Tone From the Top: The Role of the General Counsel in the Prevention of Harassment and Abuse in International Sports

**DOI:** 10.3389/fspor.2021.625684

**Published:** 2021-04-20

**Authors:** Andrea Carska-Sheppard, Sarah Ammons

**Affiliations:** ^1^General Counsel, Workplace Options LLC, Raleigh, NC, United States; ^2^Principal, Sports Law Solutions, Prague, Czechia; ^3^School of Law, University of North Carolina at Chapel Hill, Chapel Hill, NC, United States

**Keywords:** leadership, harassment, abuse, safe, sports, safeguarding, directors, prevention

## Abstract

To prevent harassment and abuse in sports, the consensus is that an essential factor is “tone from the top” of the organization. It is key as a catalyst and sustainer of change, whether in the corporate or sports world. An organization's general counsel is one of the go-to top advisors for executive leadership regarding laws and regulations. Additionally, they serve as advisors for issues in other areas, such as public policy, ethical and legal risks, and human rights. With their leadership, general counsels can play a vital role in the prevention of harassment and abuse in an organization. The guidance and leadership of the general counsel should facilitate reviewing and strengthening of the organization's policies and procedures and other strategies helping to prevent and address issues of harassment and abuse. Legal issues become more complex the more global the organization, so more complex strategies are needed to tackle these issues successfully.

We are living in times that call for great resilience, respect for human rights, and respect for ethical values. Sports, and in particular international sports, mirror social developments in our society and help set trends. To prevent harassment and abuse, the consensus is that an essential factor is “tone from the top” of the organization. It is key as a catalyst and sustainer of change, whether in the corporate or sports world.

An organization's general counsel, chief legal officer or legal director is one of the go-to top advisors for executive leadership regarding laws and regulations. Additionally, they serve as advisors for issues in other areas, such as public policy, ethical and legal risks, and compliance. With their influence, general counsels can play a vital role in the prevention of harassment and abuse in an organization. Under their guidance and leadership, the organizations can strengthen their legal frameworks and strategies that help to prevent and address issues of harassment and abuse. Legal issues become more complex the more global the organization and more complex strategies are needed to tackle these issues successfully.

This article represents the opinions of the authors and is inspired by the current initiatives dedicated to the prevention of harassment and abuse in sports (“PHAS”) including the leadership and work^2^ of the International Olympic Committee and its commitment to keep athletes safe and guard their rights. While elite athletes are at the heart and center of many safeguarding initiatives, harassment and abuse have no borders and do not occur solely in Olympic or elite sports but in sports in general^3^. The focus of this article is on the organizational side—considering the role of general counsel, or its equivalents, such as chief legal officer or legal director, as one of the key catalysts of change within an organization in preventing harassment and abuse.

In today's world, it is important for global leaders to “connect the dots.” As such the authors are seeking to draw some helpful parallels between the sport and business world that can be used to tackle these issues. This article builds on corporate expertise and a presentation Andrea Carska-Sheppard gave a few years ago at the Global General Counsel Summit^4^ at the Intercontinental Hotel in London and her work in this area going back to the IOC Consensus Statement. With more harassment and abuse allegations being brought to light^5^ it is important that organizations handle them correctly. As sports organizations help to set trends for society, it is important to put safeguards in place to prevent future abuse from happening thus promoting good governance in sports.

## Role of General Counsel

The general counsel or chief legal officer is the most senior lawyer in the legal department of a company or other organization. The role of general counsel depends on many factors but “despite the differences in the client, the duties of a general counsel are consistent: deliver the highest possible level of legal services to the client.”^6^ The role is evolving and a “good general counsel needs to bring more than just good lawyering to the job: the general counsel adds value”^7^ and “are increasingly being asked to play a dual role of legal advocate and corporate adviser”^8^.

One of the most insightful analytical works on this subject is Ben Heineman's “*Inside Counsel Revolution*”^9^ He notes that the modern general counsel is a core member of top management who influences decisions and actions, not only about legal risks, but also on public policy and geopolitics. Leadership and being principle-based are some of the main attributes of the general counsel. The role encompasses not only being the legal advisor, but serving as a statesman (or stateswoman), or diplomat, especially when operating in a global setting and crossing boundaries of different cultures and legal systems. He writes, “in brief, the wise counselor goes beyond legal doctrine to develop-and help implement-alternative solutions to problems or issues in order to advance a broader concept of what is ‘right’ and with respect to a specific, particular problem that arises initially as legal matter. The wise counseling is one of the creative acts of the General Counsel and insider counsel.”^10^

Heineman's observations, findings, and recommendations, which redefine the traditional roles of the general counsel, are based on his experience as the General Counsel of General Electric (now Lecturer on Law at Harvard Law School), but they do not apply only to commercial businesses. He views the role of general counsel on three levels—as outstanding legal expert, wise counselor and accountable leader.^11^ This goes beyond some traditional models when the general counsel limits her/his role to that of legal expert. Heineman's insightful analysis can serve as inspiration for global general counsels, legal directors, and other top legal advisors working outside of the corporate setting and provide guidance for sports, other organizations and governing bodies. The general counsel's role includes more than just advising on the law, it encompasses a variety of responsibilities and ethical obligations. The general counsel's role is anchored in legal expertise; thus, actions need to be ethical, transparent, and not wrapped in a cloth of ambiguity. In the context of investigating harassment and abuse violations, the legal counsel needs to avoid any conflicts of interest.^12^ Ethical leadership plays a major role in how an organization is run, organized, and how members of an organization are treated.

General counsels also play a significant role in setting the tone and culture of a company. While they often do not sit on the executive boards (to preserve their independence), a major obligation for general counsels, along with executives, is to set the tone of zero-tolerance when it comes to harassment and abuse.^13^ While there are some universal principles on how global general counsels can handle matters related to harassment, each approach should be based on the applicable laws, while being tailored to the particular sector or industry. This also applies to international sports federations and similar organizations. The distinguishing features between corporate organizations and international sports federations will be discussed later in this article.

## Harassment and Abuse in International Sports

Historically, sports organizations—whether professional sports leagues, international federations, or local recreational teams—did not have robust PHAS strategies. Incidents were handled mostly within the framework of human resources or *ad hoc* tribunals based on applicable employment law on a case-by-case basis. We often hear from the survivors of harassment and abuse that many cases never came to light and were “swept under the rug.” The social scientists who pioneered researching sexual abuse in sports were often criticized. Each country was just building its own legal framework and there was no uniform approach.^14^ In 2004, the first special PHAS projects started at the IOC,^15^ and following the 2007 conference in Lausanne, the IOC adopted the first IOC Consensus Statement^16^ and the first Sexual Harassment and Abuse educational tool.^17^ Then, in 2016, the IOC adopted the Second IOC Consensus Statement^18^ and put in place the “Game-Time” PHAS framework for the Rio de Janeiro Olympic Games.^19^ This framework recognized that athletes of all ages have a right to engage in “safe sport”.^20^ “Safe Sport” is “an athletic environment that is respectful, equitable, and free from all forms of non-accidental violence to athletes”.^21^ Following this initiative, the IOC released the IOC Toolkit,^22^ FIFA released their own toolkit for the safeguarding of minors.^23^ Other organizations also began releasing their own initiatives related to PHAS,^24^ including having preventive policies and procedures, appointing safeguarding officers, holding educational programmes and seminars, and working on other PHAS initiatives, strategies and prevention tools. Just recently, the IOC launched the IOC's Safeguarding Officers Sport Certificate Initiative.^25^

Although progress has been made, there is still a need for further improvement. To foster this, general counsels need to play a more active role using their voice to champion PHAS initiatives during board and executive meetings. At the Association of Corporate Counsel General Summit in London, the Prince of Wales addressed the general counsels and called for their aid to help combat climate change.^26^ He squarely described the role of in-house counsel and general counsels as very influential in shaping organizational change. His strategy is premised on the realization that general counsels have influence with the boards and decision-makers. Also, that their voices matter in making the change in global initiatives when seeking to improve social policies and the well-being of humankind. This rings true especially when influencing change within their own organization and can be also applied to tackling harassment and abuse in sports.

## Handling Alleged Incidents

General counsels are uniquely situated to handle harassment and abuse allegations. They must be on top of the situation when incidents happen by being responsive, pro-active, and communicative with the executive team, other departments, and all stakeholders. General counsels should ensure that their organization has policies, follows due process, and there is proper case management in place, so their response is professional, well-organized, and compliant with the law. While making sure that parties to the incident are safe and the organizational measures are put in place (e.g., separating the alleged aggressor), the legal counsel needs to be transparent with the parties. Specifically, they must make it clear that they represent the organization, and not the parties to the incident, and recommend they should get their own legal counsel.

As to practical steps, *Forbes* published a comprehensive summary of fifteen key steps for companies to follow when responding to sexual harassment and discrimination allegations.^27^ *Forbes'* article notes that, while legal and policy considerations are important, effective communication is equally essential. Additionally, a team of HR, legal, and (when appropriate) communications professionals should coordinate carefully with senior management on the company's response. Further, it is important to note that allegations do not necessarily mean substantiated allegations. Confidentiality of the processes should always be assured throughout.

Along with establishing policies and communicating with senior management, general counsel should ensure the complaining party is treated with respect. Human rights and dignity must be embedded in the organizational culture and should be exemplified in how the general counsel treats the parties involved.

Timing of investigation is key to its success. General counsel should be swift to attend to an investigation, including putting protective measures in place, which may sometimes be difficult if an incident happens in different time zone. If the general counsel fails to do so, complaints that are delayed or ignored may come at high costs for the organization and show the company is inconsistent with its protection of human rights. Immediate action in the harassment and abuse investigation should also apply when deciding whether to retain outside legal counsel to handle the claims and when notifying the board of any significant allegations. The board should have the ability to provide advice on the situation, ascertain whether to report criminal offenses to the public protection agencies, and determine whether to start an independent investigation vs. an internal investigation.

The general counsel should consider different factors when determining whether to recommend an independent investigation: the people involved in the conduct and if any members of management were involved; if there were violations of criminal law (if not, then management-led investigation may be appropriate); the need for transparency and avoidance of an actual, or even perception of, conflict of interest; and the need for remediation and possibility of regulatory sanctions.

Other factors may need to be considered based on the organization. “An independent investigation is necessary where senior management … may have allegedly directed, condoned, or knew or should have known about the suspected misconduct”.^28^ An internal investigation, where there is an allegation of sexual harassment, may be conducted without involvement of the audit committee, provided that senior management was not involved in the suspected misconduct. If senior management was involved, it is important to take further precautions. According to an ACC study, “an internal investigation is often a hallmark of good corporate governance and corporate citizenship. It is integral to the board's discharge of its fiduciary duties”.^29^ Stakeholders want to know that they invest into, work for, and do business with a company that is committed to ethical and lawful business practices. Conducting independent investigations also helps to address any allegations of internal misconduct.

Regardless of whether senior management or an outside party conduct the investigation, it must be impartial and professional. Cooperation with the appropriate authorities is essential to the success of the investigation. Documents should be properly preserved, and general counsel should advise everyone in the organization to be cautious about email and other communications. It is part of the role of the general counsel to defend the organization and it is important to keep in mind that any of communication or documentation can be subject to discovery during litigation. Once a claim is made, the company must put a “legal hold” in place, making sure that any relevant documents are preserved, and not deleted or destroyed, in anticipation of potential litigation. In some jurisdictions, the failure to protect documents can be subject to the punishment by the court.

Other than working with internal stakeholders, cooperating with outside counsel, and handling the press, general counsel also has the responsibility of working closely with the insurance carriers to see whether the claim is covered by the corporation's policies. Aside from handling the complaint and investigation, each organization needs to determine the impact of the claim.^30^ For example, in determining if the claim is a single event or an on-going problem, the company needs to address the impact on the culture of the company, any remedies or insurance needed, the reaction of third parties and the public, and the impact on the company's business^31^. In doing so, each organization needs to address the lessons that need to be learned from the incident and create or update its policies and preventative strategies.

In this context, it is important to note many distinguishing features of corporations and international sports federations and how this will impact the handling of the harassment and abuse allegations. Businesses typically handle harassment and abuse claims through their human resources departments (in cooperation with the legal department and others). The affected parties are often the employees of corporation but could be also other parties, such as subcontractors, providers, or other third parties. Thus, careful and fact specific analysis must be conducted, based on the laws of the particular jurisdiction, in order to determine the responsibilities and liabilities of the affected parties.

The sports world also addresses harassment and abuse based on applicable laws, policies, and procedures but the governance structure of international sports federations is very specific. Unlike the corporate world—being made up of deliberative bodies, based on the will of members operating through independent ethics commissions, and disciplinary tribunals that are subject to arbitration.^32^ The international sports federations have their specific set of stakeholders, which include athletes, coaches, federation officials, athletes' support personnel, sponsors, agents, and fans. The effective policies and procedures need to factor all of these and other nuances and general counsel must play leadership role in moving a safeguarding agenda forward through different levels of review. The mechanism and process of safeguarding needs to be sport specific and/or tailored for a particular organization. While there are many differences between sports and business organizations, there are principles and strategies that can resonate universally.

## Some Preventive Strategies

As it was noted in the IOC Consensus Statement, there are several forms of harassment and many people who can be the target of harassment.^33^ International sports federations and other international sports organizations are typically based in one country, while the athletes and entourage regularly travel and participate in competitions around the world. The legal strategies to combat harassment and abuse in sport need to be anchored within the local legal systems, while also having a global strategy and vision in place. This local to global approach means that the general counsel should be prepared to tackle these issues on both levels while the organizations need to implement the preventive structures that are universal—addressing harassment in the competitive context and in local jurisdictions.

There is no “one size fits all” and sports organizations need to reflect their particular needs in order to best structure their preventive strategies and there are many pieces which are part of the preventive structures. One of the key tools for prevention are an organization's policies and procedures.

These policies and procedures should be used as primary tools and should include a zero-tolerance policy of harassment and abuse, definitions and examples of harassment and abuse, employees' rights to complain and protections when they do, prohibitions on retaliation, information on how to file a complaint, an investigation policy, a confidentiality policy, and a disciplinary policy.^34^ In the sports context, a helpful starting point to further develop these policies, is the IOC Toolkit, which provides a policy manual that aids sports organizations in setting up their preventive structures and is a step by step guide how to safeguard athletes and their rights. When cultivating these policies and procedures, the drafters need to make sure the policies are “living documents” that are implemented and imbedded into the organizational structure and regularly updated. Additionally, the general counsel should periodically evaluate whether the organization is following the standards set forth in the policies and assure due process and case management.^35^ The organizations can adopt one universal policy or have specific sets—for competitions and minors.^36^

The PHAS policies and procedures need to be legally accurate, user friendly and continuously updated, incorporating the role of the Safeguarding Officer in the core of the strategies. As the safety and well-being of athletes is of utmost importance to the IOC and the Olympic Movement, the recent work in this area cannot emphasize more the important role of the Safeguarding Officer. It was for first time at the Rio de Janeiro Olympic Games when the Safeguarding Officer was present at the Olympic Games in order to keep athletes safe and guard their rights. There are also new educational programmes fully dedicated to PHAS, such as the IOC's Safeguarding Officers Sport Certificate initiative.^37^ The general counsel should have knowledge of these initiatives and incorporate the role of the Safeguarding Officer into the preventive framework.

One of the key tools for prevention any general counsel should encourage is education and training for the prevention of harassment, as well as other types of trainings that raise sensitivity about these issues, such as bystander training.^38^ Education and training were among one of the main recommendations to prevent sexual harassment in sports in Europe^39^ and some jurisdictions have implemented mandatory training in this area. All sports organizations ought to take this issue seriously. The education and training must be smart, interactive, and relevant to the particular continent and culture, otherwise it may become counterproductive.^40^ In sports, there are numerous organizations now offering webinars and training. In order for the preventive strategies to succeed, it must involve the entire organization, including its leadership.

To manage reputational damage, there needs to be a plan in place for effectively handling public relations. Developing a media strategy in advance is necessary and this should be prepared long before the incident occurs as part of the organizational prevention strategy. It includes having ready an experienced spokesperson, which prevents others from speaking prematurely (any substantive comments should wait until the investigation is concluded). When executing this strategy, the general counsel should work with the communication and other teams. The same applies to insurance policies and strategies—the general counsel should make sure the organization has adequate insurance coverage and those policies cover not only home jurisdictions but provide coverage for countries to which members of the organization and their entourage travel for trainings, sports competitions, and other events.

While the governance structures of companies are based on the direct accountability of different levels of employees and managers, the governance structures of international sports federations are not as rigid and personal accountability for non-compliance should be better structured and defined.^41^ General counsel and legal directors can also play pivotal roles in this regard.

## Demonstrated Commitment From the Top

General counsels have a role in turning the CEO's or top directors' attention to prevention resources and strategies. “The cornerstone of a successful harassment prevention strategy is the consistent and demonstrated commitment of senior leaders to create and maintain a culture in which harassment is not tolerated”.^42^ When harassment claims are not handled properly, the organization can face the burden of costly damage awards, litigation costs, and adverse impacts from the harassment on employees' physical and mental health. This may further result in “absenteeism and higher medical costs,” reduction in productivity of all impacted parties, reputational harm, shareholder derivative suits, among other impacts.^43^

After Larry Nassar's crimes came to light, the sports world received a loud message on the importance of engaged and pro-active safeguarding of athletes and sports. Multi-million-dollar awards to the survivors of horrific abuses speak loud and clear about the need for zero-tolerance policies.^44^ Employers around the world can be legally responsible for sexual harassment against their employees and be liable to them for damages.^45^ In the United States, “the employer will be liable for harassment by non-supervisory employees or non-employees over whom it has control (e.g., independent contractors or customers on the premises), if it knew, or should have known about the harassment and failed to take prompt and appropriate corrective action”^46^ Liability and lawsuits are just one of the adverse impacts.

According to the recent survey of chief legal officers and general counsels, one of the risks “which keeps them up at night is reputational damage to the organization”.^47^ This heralds a significant shift in recent years when the issue of reputational purity is as important as other compliance issues for the organization, such as protection of data privacy, confidentiality, and others.

The harassment and abuse will tarnish the reputation of the organization losing the confidence of its users, such as athletes (including elite athletes who are the highest risk category when it comes to the harassment and abuse^48^), their parents or guardians, and fans. When the general counsel demonstrates that harassment claims, the prevention of harassment, and the setting of a tone of zero-tolerance are taken seriously, CEOs and HR departments will be able to avoid several, if not all, of these issues.^49^ The same applies in sports setting. The tone and demonstrated commitment of the leadership matters. The compliance with human rights and ethical standards should be an integral part of the culture of any organization.

In sports, we aim toward ideals, but we put in measures to assure and to protect “clean athletes” and “clean sports” from drug doping. Similarly, and very importantly we need to safeguard “clean athletes” and “clean sports” when it comes to harassment and abuse. The purpose of the general counsel interacting daily with chief decision makers and with the board and leadership of an organization is to create a culture of respect and inclusivity where anyone subject to harassment and abuse feels safe in reporting it.^50^

Harassment and abuse know no borders. Thus, international organizations need to tackle these issues in a culturally sensitive manner, not only in their own jurisdictions, but across the globe. In that sense, strategical general counsels need to foresee and project strategies and procedures that can be implemented beyond their home country. These strategies are needed to protect the human rights of athletes, participants, their organization, and all stakeholders. To accomplish this, leaders must send the message of a demonstrated commitment to others within the organization, as if they are part of an orchestra, that will be tuned to the same purpose, with the general counsel influential in setting the tone.

“*Let us have but one end in view: the welfare of humanity.” John Amos Comenius (1592* −*1670)*

## Data Availability Statement

The original contributions presented in the study are included in the article/supplementary material, further inquiries can be directed to the corresponding author/s.

## Author Contributions

AC-S leads the project and wrote the article. SA conducted all the background research and edited article and prepared footnotes.

## Conflict of Interest

The authors declare that the research was conducted in the absence of any commercial or financial relationships that could be construed as a potential conflict of interest.

## Footnotes

^2^See for example: Mountjoy, M., Brackenridge, C., Arrington, M., Blauwet, C., Carska-Sheppard, A., Fasting, K., et al. (2016). International Olympic Committee consensus statement: harassment and abuse (non-accidental violence) in sport. *Br. J. Sports Med*. 50, 1019–1029. doi: 10.1136/bjsports-2016-096121; Safeguarding Athletes From Harassment and Abuse in Sport: IOC Toolkit for IFs and NOCs. International Olympic Committee (“IOC Toolkit”). Available online at: https://www.olympic.org/athlete365/courses/safeguarding-athletes-from-harassment-and-abuse/

^3^See for example. Emily Kaplan, Daniel Carcillo leads class-action lawsuit against Canadian Hockey League for alleged physical and sexual abuse, ESPN, Jun. 18, 2020; Jessica Luther, Jon Wertheim, A New Mavericks #MeToo Accusation-and Questions About the Team's Investigation, Sports Illustrated, Jul. 29, 2020; Scott Cacciola, Mavericks and Mark Cuban Sanctioned by N.B.A. Over Handling of Sexual Harassment, NY Times, Sept. 19, 2018; Ken Belson, N.F.L. Takes Over Sexual Harassment Investigation of Washington Team, NY Times, Sept. 1, 2020; Sports Impacted by Sexual Abuse Cases, Fox Sports, Mar. 16, 2018.

^4^The Association of Corporate Counsel organized the summit to attract chief legal counsels from global organizations to discuss the hot legal, compliance, and ethical values of the day, as well as to find solutions in a globalized world. At this event, Andrea discussed the role that the leadership of organization, such as executive boards and C-suites, can play in the prevention of harassment and abuse. The experiences she shared stemmed from her work as General Counsel of the world's largest integrated corporate wellbeing company, Workplace Options, LLC (“WPO”), which operates in 200 countries and territories while serving over 100,000 organizations, including Fortune 500 companies and sports organizations, offering employee wellbeing services including clinical counseling for harassment and abuse.

^5^North, A. (2019). *Study: More People Reported Sex Crimes Around the World in the Wake of Me Too*. Vox. A study covering 24 countries “found that the rise of the Me-Too movement led to a 14% increase in the reporting of sex crimes in the 3 months after October 2017. Reporting rates dropped after that point, but still remained above their pre-2017 levels, even 15 months after the movement began to grow.”

^6^Association of General Counsel, The Role of General Counsel.” 2009 at 7.

^7^*Ibid at 8*.

^8^Rosen, R. E. (2002). We're all consultants now: how change in client organizational strategies influences change in the organization of corporate legal services. *Ariz. Law Rev*. 44:637.

^9^Heineman, B. W. Jr. (2014). *The Inside Counsel Revolution: Resolving the Partner-Guardian Tension, 1st Edn*. Chicago, IL: ABA Publishing; American Bar Association.

^10^*Ibid at 37*.

^11^*Ibid at 32*.

^12^Some jurisdictions require legal counsel to give an “Upjohn Warning.” An Upjohn Warning is a notice that an attorney (in-house or outside counsel) provides to an employee of the company stating that the attorney represents only the company and not the employee individually. Even if the jurisdiction does not have special warnings as part of their legal system, bar organizations and licensing bodies regulate the ethical rules that bind lawyers, and lawyers should adhere to a high level of ethics.

^13^Newman, P. K. *Practical Advice for Handling Sexual Harassment Complaints in The Post–#MeTooWorld*. The Newman Law Group LLC. Available online at: https://assets.corporatecompliance.org/Portals/1/PDF/Resources/past_handouts/Regional/2018/columbus/945-1045_newman-shumate_3.pdf

^14^For example, the United States government enacted Title IX (20 U.S.C. § 1681) (1972), as part of the Education Amendments of 1972, in order to protect students from sex discrimination under any education programme or activity receiving federal funding. Though there are many critics of Title IX, this legislation looked toward the future and provided a safeguard for student athletes that some other countries did not have.

^15^*See generally Athlete 365* at https://www.olympic.org/athlete365/library/safe-sport/.

^16^Ljungqvist, A., Mountjoy, M., and Brackenridge, C. H. (2008). International olympic committee consensus statement on sexual harassment & abuse in sport. *Int. J. Sport Exerc. Psychol*. 6, 442–449.

^17^Press Release and International Olympic Committee (2007). *IOC Adopts Consensus Statement on Sexual Harassment and Abuse in Sport*. Available online at: https://www.olympic.org/news/ioc-adopts-consensus-statement-on-sexual-harassment-and-abuse-in-sport.

^18^Mountjoy, *supra*, note 2.

^19^IOC (2016). *IOC Framework for Safeguarding Athletes and Other Participants from Harassment and Abuse in Sport (Games Time Period)*. London: BMJ Publishing Group Limited.

^20^Mountjoy, *supra*, note 2.

^21^Mountjoy, *supra*, note 2.

^22^IOC Toolkit, *supra*, note 2.

^23^FIFA Guardians (2019). *Child Safeguarding Toolkit for Member Associations*. Zurich: FIFA Guardians.

^24^United Nations Human Rights Special Procedure (2019). *Playing It Safe: A Glimpse of the Report of the UN Special Rapporteur on the sale and sexual exploitation of children*. Geneva: United Nations Human Rights Special Procedure.

^25^Press Release and International Olympic Committee (2020). *IOC Initiates International Safeguarding Officer in Sport Certificate*. Available online at: https://www.olympic.org/news/ioc-initiates-international-safeguarding-officer-in-sport-certificate.

^26^Spiezio, C. (2019). *Prince Charles to General Counsel: Help Companies Combat Climate Change*. London: Law.com - Corporate Counsel.

^27^Hermle, L. (2017). *15 Key Steps for Companies Responding to Sexual Harassment or Discrimination Allegations*. Forbes. The list of steps include: (1) Lawyer Up; (2) Notify the board of directors promptly of significant allegations; (3) Treat the complaining party with respect; (4) Promptly and thoroughly investigate the complaint; (5) Take appropriate action during and after investigation; (6) If the company doesn't have a well-drafted anti-harassment and anti-discrimination policy, adopt one; (7) Cooperate with government agencies; (8) Consider whether the complaint can be resolved through arbitration; (9) Don't retaliate; (10) Be careful with texts and email; (11) Preserve Documents; (12) Make Claim to your Insurance broker; (13) Develop a Media Strategy; (14) Assess the Impact; (15) If you settle, include these provisions in a settlement agreement.

^28^Hicks, K. L., Bondi, B. J., Wheatley, M. D. (2016). *When Should the General Counsel Recommend That the Board Conduct an Independent Investigation?* Washington, DC: ACC Docket.

^29^*Id*.

^30^Hermle, *supra*, note 27.

^31^*Id*.

^32^The discussion on the harassment and abuse and dispute resolution mechanisms were discussed for example at Yale Law School co-organized a virtual roundtable on this topic. *See #MeTooSport in the Larry Nassar Era: Legal Frameworks for Preventing Intentional Violence in Sport Roundtable*, Dec. 2, 2020.

^33^See Mountjoy, *supra*, note 2. The “IOC Consensus Statement” outlined various forms of abuse and harassment in sports, showing strong evidence of a correlation between sexual, physical, and psychological abuse. It analyzed various mechanisms for Harassment and Abuse and was one of the first to recognize that the risks to young athletes are unique to sport and dealt with the issue of consent with minors.

^34^Hermle, *supra*, note 27; Newman, *supra*, note 13.

^35^Brown, K., and Ellis, J. (2019). *5 Strategies for In-house Counsel to Enhance Their Organization's Workplace Culture in the Era of #MeToo*. Philadelphia, PA: Duane Morris.

^36^There is significant focus these days on the policies and procedure but drafting of these is complex and is beyond the scope of this article.

^37^Press Release, *supra*, note 25.

^38^Smith, B. L. (2018). *What It Really Takes to Stop Sexual Harassment*. Washington, DC: American Psychological Association.

^39^Chroni, S., Fasting, K., Hartill, M., Knorre, N., Martin, M., Papaefstathiou, M., et al. (2012). *Prevention of Sexual and Gender Harassment and Abuse in SPORTS INITIATIVES in Europe and Beyond, Prevention of Sexualised Violence in Sports*. Frankfurt: Deutsche Sportjugend im Deutschen Olympischen Sportbund e.V.

^40^Dobbin, F., and Kalev, A. (2020). *Why Sexual Harassment Programs Backfire*. Brighton, MA: Harvard Business Review.

^41^Pielke, R. Jr. (2016). *Obstacles to Accountability in International Sports Governance*. Transparency International. Available online at: https://www.transparency.org/files/content/feature/1.4_ObstaclesToAccountability_Pielke_GCRSport.pdf.

^42^U.S. Equal Employment Opportunity Commission (2017). *Promising Practices for Preventing Harassment*. Washington, DC: U.S. Equal Employment Opportunity Commission.

^43^Feldblum, C. R., and Masling, S. P. (2018). *Convincing CEOs to Make Harassment Prevention a Priority*. Brighton, MA: Harvard Business Review.

^44^See generally Phillips, F. (2020). *Sexual Harassment Payouts Hit All-Time High in 2019*. Fisher Phillips. “Employers paid out a record $68.2 million to those alleging sexual harassment violations through the EEOC in 2019, shattering the all-time record by over $10 million and reminding us all that the #MeToo movement continues to be a major influence on workplaces across the country.”

^45^“Employer Liability for Harassment,” U.S. Equal Employment Commission; See also Gregory, H. J. (2018). *Board Oversight of Sexual Harassment Risk in the Workplace*. New York, NY: The Governance Counselor.

^46^“Harassment,” U.S. Equal Employment Commission.

^47^Association of Corporate Counsel (2019). *2019 ACC Chief Legal Officers Survey*. Washington, DC: Association of Corporate Counsel.

^48^Mountjoy, *supra*, note 2. “This consensus statement extends the 2007 IOC Consensus Statement on Sexual Harassment and Abuse in Sport, presenting additional evidence of several other types of harassment and abuse-psychological, physical and neglect. All ages and types of athletes are susceptible to these problems, but science confirms that elite, disabled, child and lesbian/gay/bisexual/trans-sexual, LGBT athletes are at highest risk, that psychological abuse is at the core of all other forms and that athletes can also be perpetrators.”

^49^See Feldblum, *supra*, note 43.

^50^See Smith, supra, note 38.

